# Lifestyle factors and urine levels of organophosphorus flame retardants in endometrial cancer: insights from a case-control study

**DOI:** 10.1265/ehpm.24-00175

**Published:** 2024-11-09

**Authors:** Yu-Che Ou, Fu-Jen Cheng, Wan-Ting Huang, Wen-Chin Lee, Hung-Chun Fu, Chen-Hsuan Wu, Ying-Yi Chen, Kuo-Chung Lan, Xuan-Ping Liou, Hao Lin, Chia-Te Kung

**Affiliations:** 1 Department of Obstetrics and Gynecology, Kaohsiung Chang Gung Memorial Hospital and Chang Gung University College of Medicine, Kaohsiung, Taiwan; 2 Department of Obstetrics and Gynecology, Chiayi Chang Gung Memorial Hospital and Chang Gung University College of Medicine, Chiayi, Taiwan; 3 Department of Emergency Medicine, Kaohsiung Chang Gung Memorial Hospital and Chang Gung University College of Medicine, Kaohsiung, Taiwan; 4 Department of Laboratory Medicine, Kaohsiung Chang Gung Memorial Hospital and Chang Gung University College of Medicine, Kaohsiung, Taiwan; 5 Division of Nephrology, Department of Internal Medicine, Kaohsiung Chang Gung Memorial Hospital and Chang Gung University College of Medicine, Kaohsiung, Taiwan; 6 Department of Emergency Medicine, Jen-Ai Hospital, Taichung, Taiwan

**Keywords:** Organophosphate flame retardants, Lifestyle behavior, Endometrial cancer

## Abstract

**Background:**

Organophosphate flame retardants (OPFRs) are commonly used in various consumer products to prevent fire hazards. However, OPFRs have been linked to several health problems, including cancer. This study aimed to investigate the association between urine levels of OPFRs and endometrial cancer (EC), and to explore the correlation between concentrations of parent OPFR compounds and their metabolites.

**Methods:**

Urine samples from 76 EC patients and 76 healthy controls were collected and analyzed for the levels of five common parent OPFRs and their respective metabolites. Propensity score matching was applied to account for differences in baseline characteristics between the two participant groups. Significantly higher levels of OPFRs in EC patients were identified, and logistic regression models were used to determine whether elevated OPFRs were associated with EC and to explore whether any lifestyle behaviors contributed to the increased OPFR levels. Spearman’s rank correlation coefficients between the concentrations of the parent compounds and their metabolites were calculated.

**Results:**

Out of the ten OPFRs studied, the median urine levels of bis(1,3-dichloro-2-propyl) phosphate (BDCPP), tris(2-butoxyethyl) phosphate (TBEP), and di-(2-butoxyethyl) phosphate (DBEP) were significantly higher in EC patients compared to healthy controls. After matching 41 patients with 41 controls, multiple logistic regression analysis revealed that only BDCPP (OR 4.274; 95% CI 1.172–15.592) was an independent factor associated with EC. A lifestyle questionnaire survey found that urine BDCPP levels were related to age (OR 4.294; 95% CI 1.015–18.164), meals eaten out (OR 4.238; 95% CI 1.454–12.354), and consumption of chilled-ready meals (OR 0.118; 95% CI 0.014–0.985). A positive correlation was only observed between the concentrations of TBEP and its metabolite DBEP; other correlations were not significant.

**Conclusion:**

We concluded that higher urine BDCPP level was an independent factor associated with EC, and higher BDCPP levels were related to aging, more meals eaten out, and fewer chilled-ready meals. These findings highlight the potential hazard of long-term OPFR exposure on the development of EC.

**Supplementary information:**

The online version contains supplementary material available at https://doi.org/10.1265/ehpm.24-00175.

## Introduction

Flame retardants are chemicals that have been widely applied in various products such as furniture, electronics, and building materials for slowing or preventing the spread of fire. For example, polybrominated biphenyls were used in products until they were phased out in 1976. They were then replaced until the early 2000s by a group of very similar chemicals called polybrominated diphenyl ethers (PBDEs). According to the National Cancer Institute, PBDEs may induce cancers in animal studies [1]. The capability of PBDEs to accumulate in human tissue and breast milk has raised concerns about their potential impact [2]. Therefore, other flame retardants like organophosphate flame retardants (OPFRs) have emerged as alternatives to PBDEs. OPFRs are used in other applications such as food packaging materials, plasticizers or lubricants for fire safety [3].

The toxicity of OPFRs has been greatly reduced due to recent global progress in the management, disposal, and recycling of OPFRs. However, many OPFRs are additives and not chemically bonded to the final product; therefore, significant amounts of OPFRs are released into the surrounding environment through volatilization, leaching, attrition, and dissolution of polymeric materials. OPFR has been detected in various environments, including indoor and outside air, drinking water, dust, and soil [4, 5]. Several studies have found that OPFR exposure can affect the levels and activity of hormones such as estrogen, testosterone, and progesterone, which play important roles in reproductive health and development [6–8].

Endometrial cancer is the most common gynecological cancer in developed countries with a rapidly increased global incidence. Over the past 30 years, the overall incidence has risen by 132% [9]. Endometrial cancer is considered as hormone-dependent tumor. Long-term exposure to estrogen, for example, through early menarche, late menopause, anovulation, obesity, or the use of unopposed estrogen therapy are the main risk factors [10]. Carcinogenesis requires several different mechanisms and factors, including genetic and environmental (chemicals, radiation, viruses). OPFRs and their metabolites affect hormone profile interactions and have been found in human samples, thus may contribute to hormone-dependent carcinogenesis. Therefore, this study was aimed to assess concentrations of common OPFRs and their respective metabolites in urine and to analyze the associations between OPFRs levels and endometrial cancer risk, as well as the correlation between concentrations of parent OPFR compounds and their metabolites. We also attempted to analyze the impact of different lifestyle-related behaviors on OPFRs levels through questionnaire survey.

## Methods

### Study populations

This study was conducted at Kaohsiung Chang Gung Memorial Hospital, Kaohsiung, Taiwan, with approval from the institutional ethics committee (No. 202001030B0) and informed consent obtained from each participant. We enrolled participants based on their availability and willingness during the study period. Patients with pathologically proved EC were recruited from two specialist gynecologic oncologists (Y. C. Ou and H. Lin) into the study group from the outpatient clinics between October 2020 and January 2022. The inclusion criteria were as follows: (1) being adult patients not younger than 20 years old; (2) having pathologically confirmed EC of any histologic type; and (3) being willing to participate in the study and compliance with the study protocol. Women who were undergoing chemotherapy or radiotherapy, drug-abuser, and active pregnancy were excluded from the study. Healthy women, without a history of any type of cancer or endometrial hyperplasia, were recruited from family members of the study cases, hospital staff, and community women. We made efforts to recruit the control group from multiple sources and ensured that the health status of these participants accurately reflected that of the general population. These participants were routinely asked about any gynecological diseases during the recruitment process to ensure they met health standards. All members of the control group were required to adhere to the same compliance standards as the cases.

### Lifestyle questionnaire survey

All recruited eligible women were required to complete a comprehensive questionnaire that collected information on their demographics, lifestyle-related behaviors, and the eating habits. A clinical research associate checked the survey form for completion and aided if needed. The survey form consisted of 15 questions including 1) age; 2) body mass index; 3) long-term medication; 4) chronic disease; 5) family history of cancer; 6) family history of chronic disease; 7) alcohol drinking behavior defined as seldom (almost not or occasionally) or often (at least one cup in daily); 8) meals eaten out per week defined as ≤ or >7 times; 9) regular handwashing defined as seldom (almost not or occasionally before meal) or often (almost or must before meal); 10) use of plastic food containers defined as seldom (almost not or occasionally in daily) or often (at least one times in daily); 11) frequent chilled-ready meals consumption defined as seldom (almost not or occasionally in daily) or often (at least one times in daily); 12) urinate times daily defined as ≤ or >5 times; 13) frequent seafood meals consumption defined as seldom (almost not or occasionally in daily) or often (at least one times in daily); 14) frequent meat meals consumption defined as seldom (almost not or occasionally in daily) or often (more than 0.5 palm size in daily); 15) occupational exposures to OPFRs defined as low risk or high risk (working in furniture, electronics, textile manufacturing, recycling facilities, or firefighters). The lifestyle questionnaire, with detailed definitions of each variable, is provided in the supplementary file A.

### Urine samples collection

Urine samples for the disease group were collected immediately after EC diagnosis to establish baseline data, while those for the control group were collected shortly after informed consent. Spot urine samples (n = 152) were collected using a polypropylene plastic cup (409726, Deltalab, Barcelona, Spain) stored in 1.5 ml amber microcentrifuge tubes (TUBE-170-A, ExtraGene, Taichung, Taiwan) and kept at −80 °C until analysis.

### Sample preparation and solid-phase extraction

A 200 µL aliquot of urine was adjusted to pH 6.5 and treated with 20 µL of β-glucuronidase enzyme, followed by mixing with 50 µL of 250 ng/mL isotope-labeled internal standards. After incubating at 37 °C for 2 hours, the urine samples were transferred to a solid-phase extraction (SPE) plate for analyte extraction and concentration. The SPE cartridges were pre-conditioned with 1 mL of methanol and 1 mL of Milli-Q water, and the analytes were eluted with 250 µL of 0.5% formic acid in acetonitrile.

### Analysis of OPFRs

Five common OPFRs (begin with letter T): tris(1,3-dichloro-2-propyl) phosphate (TDCPP), tris(2-butoxyethyl) phosphate (TBEP), tri(2-chloroethyl) phosphate (TCEP), tri-n-butyl phosphate (TnBP), triphenyl phosphate (TPhP)), and their respective metabolites (begin with letters B or D): bis(1,3-dichloro-2-propyl) phosphate (BDCPP), di-(2-butoxyethyl) phosphate (DBEP), bis(2-chloroethyl) hydrogen phosphate (BCEP), di-n-butyl phosphate (DnBP), diphenyl phosphate (DPhP), were included for analysis.

The analysis methods for organophosphate flame retardants (OPFRs) in this study were adapted from a previous study [11]. In brief, all OPFRs and their metabolites were separated using a Waters Acquity Ultra-Performance Liquid Chromatography (UPLC) BEH Phenyl column followed by a Waters XBridge BEH C18 Direct Connect HP isolated column. The mobile phases consisted of 0.5% formic acid in water (A) and 0.5% formic acid in methanol (B). The gradient was increased linearly from an initial 5% to 50% of solvent (B) within 0.75 min. Then, the mobile phase (B) was increased to 100% in another 3 min and held for 4.5 min. Finally, the gradient was decreased to the initial 5% of solvent (B) for a 2 min re-equilibration. The target analytes were identified based on their retention time and ratio of the two selected precursor ion-produced ion transitions compared to those of the standards. The concentrations of OPFRs were quantified using 12-point calibration curves (ranging from 0.02 ppb to 50 ppb with a two-fold dilution), and their recoveries were performed using the internal standard method based on individual isotope-labeled internal standards. The internal standards, including TnBP-d27, DNBP-d4, TCEP-d12, BCEP-d8, TBEP-d27, DBEP-d4, TPHP-d15, DPHP-d10, TDCPP-d15 and BDCPP-d10 were purchased from Toronto Research Chemicals Inc. (Toronto, ON, Canada). Detailed information on the substances used is provided in supplementary file B. We defined the peak limits of detection (LOD) as a signal-to-noise ratio greater than 3 and the limits of quantification (LOQ) as a signal-to-noise ratio greater than 10. The current LOQ was set at 0.001 ppb. All the calibration curves provided adequate linearity (R2 > 0.995) with a validated analytical range. Each result for an OPFR compound was corrected using the urinary creatinine level of the corresponding urine sample.

### Statistical analyses

Because there was potential for Berkson’s bias in participants selection process, propensity score matching (PSM) was performed to balance the disease and control groups. PSM was conducted using nearest neighbor matching with a 0.02 caliper to address imbalance. Conditional logistic regression analysis was used to adjust for confounding factors and assess the relationship between disease and control groups. Covariate imbalance in unmatched and matched samples was measured using standardized mean differences (SMD). A SMD of less than 0.1 indicates negligible differences; 0.1 to 0.3, small differences; 0.3 to 0.5, moderate differences; and above 0.5, significant differences. Percentage distributions were used to analyze the descriptive characteristics of the subjects. Means and standard deviations (SD) or medium and interquartile range (IQR) were used to calculate the continuous variables. Receiver operating characteristic (ROC) curve analysis was used to identify optimal cut-point levels of OPFRs by determining the point with maximal Youden index (sensitivity + specificity − 1). Comparisons of median and mean values were performed using the Mann-Whitney U test. Percentage distributions between categorical variables were compared using the chi-square test. Univariate and multivariate logistic regression analyses were used to calculate the odds ratios (ORs) and corresponding 95% confidence intervals (CIs) to reflect the impact of different variables on EC or OPFRs level. Spearman’s correlation test was used to exam whether two variables are correlated. Statistical analysis was performed using SPSS statistical software for Windows version 21 (SPSS for Windows, version 21). A *P* value less than 0.05 was considered statistically significant.

## Results

Initially, 201 women were assessed for eligibility during the study period, including 116 cases in the disease group and 85 in the control group. After excluding those who were unwilling to participate, a total of 152 women were included in the final analysis, with 76 women in each group. In disease group, 59 cases were stage I, seven cases were stage II, while nine and one cases were stage III and IV, respectively. All participants’ lifestyle-related behaviors were investigated based on the contents of questionnaire. Compared to the control group, the disease group had a higher proportion of patients who were over 40 years old, had a BMI greater than 24, used long-term medication, had chronic diseases, and were exposed to occupational OPFRs. After PSM, 41 cases from each group were included. The results indicated that the baseline characteristics between the disease and control groups were well-balanced, with nearly all SMD below 0.1. Detailed demographic profiles of study population between control and disease groups before and after PSM are shown in Table 1.

**Table 1:** Demographic profiles of control and disease groups before and after propensity score matching.

	**Before propensity score matching**			**After propensity score matching**		
	
**Clinical characteristics**	**Control N = 76** **n (%)**	**Disease N = 76** **n (%)**	**SMD**	**Control N = 41** **n (%)**	**Disease N = 41** **n (%)**	**SMD**
Age (years)						
≤40	21 (27.63)	10 (13.16)		4 (9.76)	5 (12.19)	
>40	55 (72.37)	66 (86.84)	0.365	37 (90.24)	36 (87.81)	0.078
Body mass index (BMI)						
≤24	51 (67.11)	35 (46.05)		22 (53.66)	24 (58.54)	
>24	25 (32.89)	41 (53.95)	0.435	19 (46.34)	17 (41.46)	0.098
Family history of cancer						
No	42 (55.26)	32 (42.10)		18 (43.90)	15 (36.58)	
Yes	34 (44.74)	44 (57.90)	0.266	23 (56.10)	26 (63.42)	0.150
Family history of chronic disease						
No	29 (38.16)	23 (30.26)		14 (34.15)	13 (31.71)	
Yes	47 (61.84)	53 (69.74)	0.167	27 (65.85)	28 (68.29)	0.052
Chronic medication^a^						
No	40 (52.63)	16 (21.05)		11 (26.83)	10 (24.39)	
Yes	36 (47.37)	60 (78.95)	0.693	30 (73.17)	31 (75.61)	0.056
Chronic disease^b^						
No	54 (71.05)	36 (47.37)		24 (58.54)	25 (60.98)	
Yes	22 (28.95)	40 (52.63)	0.497	17 (41.46)	16 (39.02)	0.050
Occupation exposure^c^						
Low risk	42 (55.26)	21 (27.63)		19 (46.34)	19 (46.34)	
High risk	34 (44.74)	55 (72.37)	0.584	22 (53.66)	22 (53.66)	<0.001

SMD: standardized mean difference, less than 0.1 indicates very small differences; values between 0.1 and 0.3 indicate small differences; values between 0.3 and 0.5 indicate moderate differences; values above 0.5 indicate considerable differences.

^a^Chronic medication: 56 subjects without taking medicine, 53 subjects with taking prescription medicines, 43 subjects with taking health supplements.

^b^Chronic disease: 90 subjects without chronic disease, 54 subjects with common chronic diseases (diabetes, high blood pressure, hyperlipidemia, heart disease, kidney disease, chronic liver disease), 8 subjects with others (allergies, thalassemia, gallstones, thyroid disease).

^c^Occupation: 63 subjects with low-risk exposure (unemployed, retire, civilian staff), 89 subjects with high risk (semiconductor industry, traditional industry, biochemistry laboratory, environmental cleaner, night market employees, civil engineering, agriculture).

**Fig. 1:**
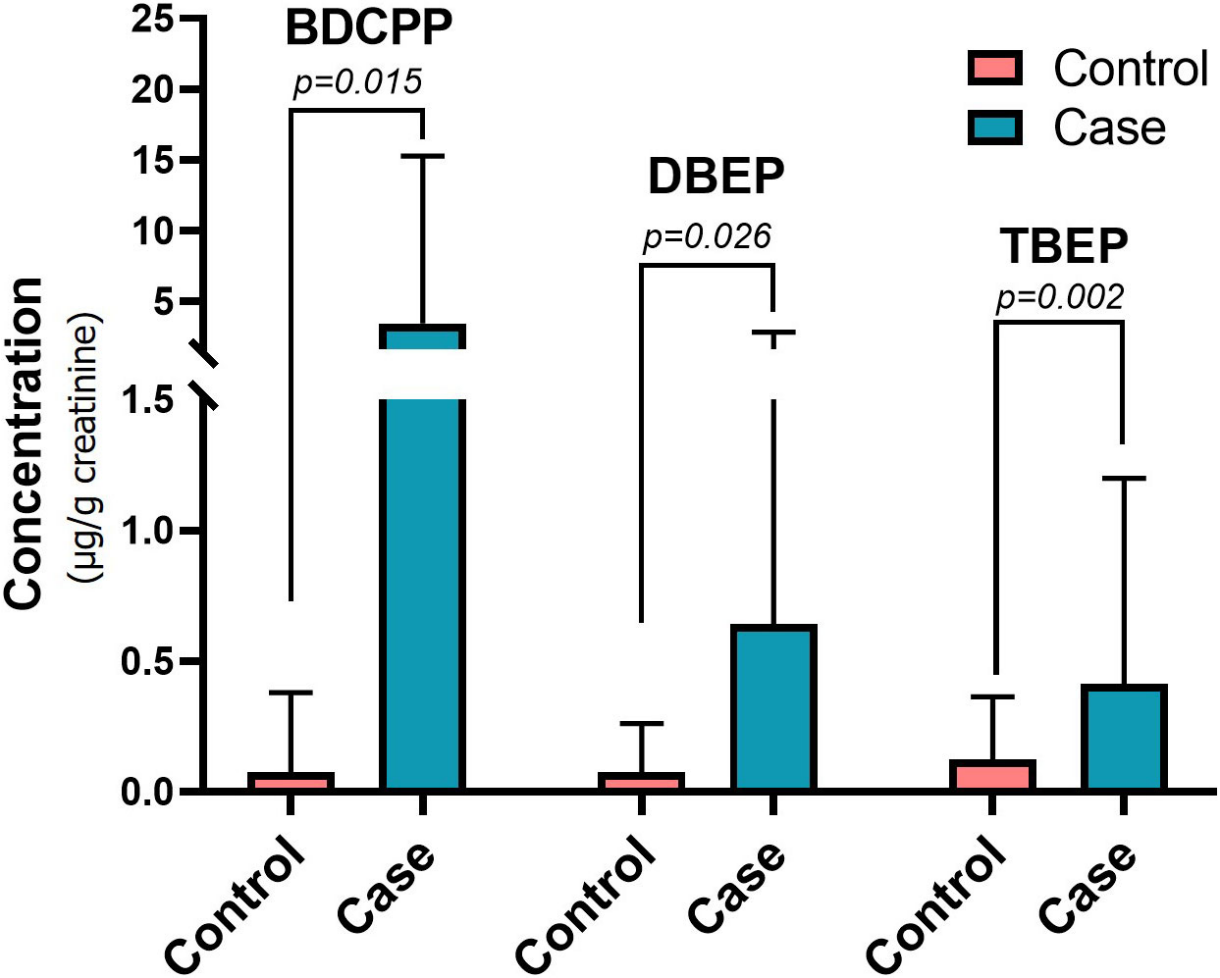
Bar plots of average BDCPP, DBEP, and TBEP concentrations in control and disease groups.

With UPLC-MS/MS, 5 OPFRs and their metabolites in 152 participants were analyzed and presented as mean value with SD and median value with IQR between control and disease groups as listed in Table 2. Among various OPFRs and their metabolites, the mean and median concentrations of BDCPP, DBEP, and TBEP were significantly higher in the disease group while no difference was observed for others (Fig. 1). The next step was attempted to determine an adequate cut-off concentration of each of these three OPFRs to predict EC. The ideal cut-off value for BDCPP, DBEP, and TBEP was 0.505, 0.106, and 0.085 µg/g creatinine, respectively (Table 3A). Based on these cut-off values, we found that significantly more patients in the disease group had higher BDCPP, DBEP, and TBEP levels (Table 3B).

**Table 2:** Comparison of mean and median OPFR concentrations between control and disease groups.

**OPFRs and metabolites**	**Control (N = 76)**		**Disease (N = 76)**		***P*-value (mean difference)**
	Mean ± SD	Medium (IQR range)	Mean ± SD	Medium (IQR range)	
BDCPP (µg/g creatinine)	0.075 ± 0.305	0.000 (0.000–1.770)	3.458 ± 11.834	0.000 (0.000–95.280)	0.015
BCEP (µg/g creatinine)	0.148 ± 0.367	0.000 (0.000–2.116)	0.816 ± 3.007	0.000 (0.000–24.716)	0.055
DBEP (µg/g creatinine)	0.076 ± 0.186	0.000 (0.000–1.133)	0.642 ± 2.203	0.137 (0.000–18.514)	0.026
DnBP (µg/g creatinine)	0.011 ± 0.034	0.000 (0.000–0.190)	0.005 ± 0.019	0.000 (0.000–0.110)	0.126
DPhP (µg/g creatinine)	0.220 ± 0.424	0.090 (0.000–3.280)	0.776 ± 4.402	0.155 (0.000–38.420)	0.276
TDCPP (µg/g creatinine)	0.028 ± 0.091	0.000 (0.000–0.530)	0.030 ± 0.107	0.000 (0.000–0.630)	0.999
TCEP (µg/g creatinine)	0.118 ± 0.502	0.000 (0.000–2.860)	0.243 ± 0.853	0.000 (0.000–3.620)	0.304
TBEP (µg/g creatinine)	0.124 ± 0.239	0.002 (0.000–1.070)	0.412 ± 0.787	0.115 (0.000–3.960)	0.002
TnBP (µg/g creatinine)	0.041 ± 0.055	0.019 (0.000–0.290)	0.040 ± 0.047	0.030 (0.000–0.230)	0.880
TPhP (µg/g creatinine)	0.015 ± 0.033	0.000 (0.000–0.230)	0.014 ± 0.029	0.000 (0.000–0.180)	0.765

A *P* values less than 0.05 is considered as statistically significant.

**Table 3:** OPFRs cut-point values (A) and distribution of OPFR levels in control versus disease groups (B).

**A**					

**OPFRs and metabolites**	**Cut-point value**	**Sensitivity**	**1-specificity**	**Youden’s index**	***P*-value**
BDCPP (µg/g creatinine)	0.505	0.408	0.053	0.355	0.003
BCEP (µg/g creatinine)	0.588	0.276	0.079	0.197	0.392
DBEP (µg/g creatinine)	0.106	0.579	0.197	0.382	0.000
DnBP (µg/g creatinine)	0.001	0.895	0.724	0.171	0.085
DPhP (µg/g creatinine)	0.425	0.197	0.092	0.105	0.457
TDCPP (µg/g creatinine)	0.565	0.026	0.000	0.026	0.117
TCEP (µg/g creatinine)	3.0100	0.066	0.000	0.066	0.136
TBEP (µg/g creatinine)	0.085	0.553	0.263	0.289	0.004
TnBP (µg/g creatinine)	0.019	0.776	0.500	0.276	0.322
TPhP (µg/g creatinine)	0.065	0.079	0.053	0.026	0.415

**B**			

**OPFRs and metabolites**	**Control N = 76** **n (%)**	**Disease N = 76** **n (%)**	***P*-value**
BDCPP			
Low	72 (94.74)	45 (59.21)	
High	4 (5.26)	31 (40.79)	<0.001
DBEP			
Low	61 (80.26)	32 (42.11)	
High	15 (19.74)	44 (57.89)	<0.001
TBEP			
Low	56 (73.68)	34 (44.74)	
High	20 (26.32)	42 (55.26)	<0.001

We further investigated whether these three OPFRs were independent factors associated with EC while other potential clinical factors were also taken into consideration. Multiple logistic regression analysis of the 152 study participants showed that BDCPP (OR 10.565; 95% CI 3.045–36.651, *p* < 0.001), DBEP (OR 3.610; 95% CI 1.406–9.264, *p* = 0.008), long-term medication (OR 2.973; 95% CI 1.159–7.624, *p* = 0.023), and occupation (OR 3.468; 95% CI 1.361–8.835, *p* = 0.009) were significant independent factors associated with EC (Table 4A). However, after adjusting for age, BMI, chronic medication use, chronic disease, occupation, and family history of cancer and chronic disease in a cohort of 82 matched participants, multivariate logistic regression analysis identified BDCPP as the only independent predictor of EC (adjusted OR 4.274; 95% CI 1.172–15.592, *p* = 0.028) (Table 4B).

**Table 4:** Results of univariate and multivariate logistic regression analyses in the prediction of endometrial cancer

**A. Before propensity score matching**						

**Variables for predicting ** **endometrial cancer**	**Univariate analysis**			**Multivariate analysis**		

	**OR**	**(95% CI)**	***P*-value**	**OR**	**(95% CI)**	***P*-value**
Age	2.520	1.095–5.801	0.030	1.134	0.291–4.410	0.856
BMI	2.390	1.238–4.612	0.009	2.508	0.873–7.207	0.088
Chronic medication	4.167	2.045–8.491	0.000	4.146	1.296–13.257	0.016
Chronic disease	2.727	1.396–5.328	0.003	0.994	0.313–3.152	0.991
Occupation	3.235	1.645–6.362	0.001	2.555	0.816–8.002	0.107
Family history of cancer	1.699	0.894–3.227	0.106	2.243	0.751–6.701	0.148
Family history of chronic disease	1.422	0.725–2.789	0.306	1.211	0.321–4.565	0.778
BDCPP	12.400	4.103–37.472	0.000	11.745	2.468–55.904	0.002
DBEP	5.592	2.707–11.552	0.000	7.624	2.242–25.926	0.001
TBEP	3.459	1.749–6.841	0.000	1.302	0.379–4.480	0.675

**B. After propensity score matching**						

**Conditioner**	**After propensity score matching**					

	**Univariate analysis**			**Multivariate analysis**		

	**aOR**	**(95% CI)**	***P*-value**	**aOR**	**(95% CI)**	***P*-value**
BDCPP	5.000	1.448–17.271	0.011	4.274	1.172–15.592	0.028
DBEP	2.857	1.208–6.757	0.017	1.860	0.655–5.280	0.244
TBEP	2.600	0.927–7.293	0.069	1.820	0.518–6.397	0.350

BMI, body mass index; CI, confidence interval; OR, odds ratio

aOR: adjusted odds ratio (adjusted for age, BMI, chronic medication, chronic disease, occupation, family history of cancer and chronic disease).

Since BDCPP and DBEP were the potential OPFRs associated with EC, we then attempted to investigate if any lifestyle-related behaviors would contribute to these higher levels. Results indicated that higher urine BDCPP concentration was associated with age (OR 4.294; 95% CI 1.015–18.164, *p* = 0.048), meals eaten out (OR 4.238; 95% CI 1.454–12.354, *p* = 0.008), and chilled-ready meals (OR 0.118; 95% CI 0.014–0.985, *p* = 0.048) (Table 5A). The concentration of DBEP was found to be only marginally significant correlated with urinate habit (OR 4.042; 95% CI 1.002–16.295, *p* = 0.050) (Table 5B).

**Table 5:** Univariate and multivariate logistic regression predicting high urinary BDCPP (5A) and DBEP (5B) levels.

**A.**						

**Variables including lifestyle ** **behavior for predicting high ** **urine BDCPP level**	**Univariate analysis**			**Multivariate analysis**		

	**OR**	**(95% CI)**	***P*-value**	**OR**	**(95% CI)**	***P*-value**
Age	3.356	0.954–11.799	0.059	4.294	1.015–18.164	0.048
BMI	1.522	0.713–3.249	0.278	1.526	0.629–3.699	0.350
Alcohol drinking	1.606	0.518–4.983	0.412	2.363	0.606–9.205	0.215
Meals eaten out	2.985	1.207–7.383	0.018	4.238	1.454–12.354	0.008
Regular handwashing	0.459	0.175–1.206	0.114	0.421	0.128–1.384	0.154
Plastic container using	0.533	0.247–1.150	0.109	0.704	0.284–1.745	0.449
Chilled-ready meals	0.108	0.014–0.830	0.032	0.118	0.014–0.985	0.048
Urinate times daily	1.140	0.352–3.686	0.827	0.835	0.189–3.686	0.812
Frequent seafood	0.665	0.310–1.429	0.296	1.150	0.423–3.125	0.785
Frequent meat	0.776	0.296–2.031	0.605	0.916	0.252–3.330	0.894
Chronic medication	2.882	1.165–7.132	0.022	2.107	0.716–6.205	0.176
Chronic disease	1.511	0.706–3.232	0.287	0.708	0.267–1.880	0.488
Occupation	1.745	0.783–3.890	0.173	2.284	0.832–6.266	0.109
Family history of cancer	1.843	0.849–4.004	0.122	2.443	0.950–6.283	0.064
Family history of chronic disease	1.679	0.720–3.912	0.230	1.818	0.604–5.472	0.287

**B.**						

**Variables including lifestyle ** **behavior for predicting high ** **urine DBEP level**	**Univariate analysis**			**Multivariate analysis**		

	**OR**	**(95% CI)**	***P*-value**	**OR**	**(95% CI)**	***P*-value**
Age	1.721	0.732–4.051	0.214	1.272	0.477–3.392	0.630
BMI	1.638	0.847–3.167	0.142	1.787	0.852–3.751	0.125
Alcohol drinking	0.690	0.227–2.098	0.513	0.659	0.198–2.195	0.497
Meals eaten out	1.143	0.581–2.249	0.699	1.142	0.527–2.474	0.737
Regular handwashing	0.585	0.236–1.452	0.248	0.835	0.299–2.333	0.731
Plastic container using	0.831	0.433–1.598	0.579	1.490	0.685–3.239	0.314
Chilled-ready meals	0.317	0.113–0.896	0.030	0.360	0.119–1.092	0.071
Urinate times daily	3.879	1.078–13.954	0.038	4.042	1.002–16.295	0.050
Frequent seafood	0.748	0.383–1.460	0.395	0.749	0.328–1.710	0.493
Frequent meat	1.018	0.428–2.424	0.968	1.085	0.376–3.128	0.881
Chronic medication	2.028	1.002–4.108	0.049	1.264	0.547–2.925	0.584
Chronic disease	2.801	1.425–5.506	0.003	2.197	0.968–4.985	0.060
Occupation	1.890	0.957–3.736	0.067	1.610	0.734–3.528	0.235
Family history of cancer	0.970	0.505–1.863	0.927	0.845	0.400–1.784	0.659
Family history of chronic disease	1.491	0.739–3.010	0.265	1.254	0.521–3.022	0.613

BMI, body mass index; CI, confidence interval; OR, odds ratio

Theoretically, these five OPFRs should have a negative correlation with their corresponding metabolites. That means an increase in the urinary concentration of the parent compound should be accompanied by a corresponding decrease in the concentration of its metabolite, indicating that the metabolism of the parent compound is actively occurring. Analysis results showed that there was a significant positive correlation between the concentration TBEP and its metabolite DBEP (Spearman’s correlation coefficient 0.493, *p* < 0.001). However, no significant correlation was observed between the concentrations of other parent OPFRs and their metabolites (Table 6).

**Table 6:** Results of Spearman correlation analysis in five OPFRs with their corresponding metabolites.

**Parental OPFRs**	**Corresponding metabolites**	**Spearman correlation Coefficient (ρ)**	***P*-value**
TDCPP	BDCPP	0.074	0.363
TBEP	DBEP	0.493	<0.001
TCEP	BCEP	0.107	0.188
TnBP	DnBP	−0.018	0.829
TPhP	DPhP	0.042	0.610

OPFRs, organophosphate flame retardants

## Discussion

This study is the first one to show that women with higher urinary BDCPP concentration may be associated with EC, and this higher level was related to distinct lifestyle behavior such as aging, more meals eaten out, and fewer chilled-ready meals. Since BDCPP is the major metabolite of the parent compound TDCPP, our findings highlight the potential hazard of long-term TDCPP exposure on the development of EC. TDCPP had been reported as having tumor-promoting effects in animal studies and this chemical has been listed as a probable human carcinogen since 2011 in the United State [12, 13].

Although most OPFRs are not currently listed as carcinogens or potential carcinogens by several regulatory agencies, studies have shown associations between exposure to certain OPFRs and various types of cancer, including papillary thyroid cancer, breast cancer, cervical cancer, and gastrointestinal cancer [14–16]. However, the exact mechanisms and the extent of these associations need further investigation. A recent in vitro study suggested that OPFRs-induced activation of the PI3K/Akt/mTOR signaling pathway may be one of the possible mechanisms [17]. Furthermore, exposure to OPFRs can lead to DNA damage and cellular dysfunction [18]. In a more recent study, Ji et al. found that TDCPP disrupt hormone function by increasing estradiol synthesis in vitro [19], which may be a possible explanation for our finding.

The metabolism of OPFRs after human exposure can be complicated. Once inside the body, OPFRs can be converted into various metabolites, some of which are more toxic than the parent compounds [20]. These metabolites can then be excreted in urine or feces, or they may accumulate in fatty tissues. There have been several studies investigating a correlation between environmental levels of parent OPFRs and their corresponding metabolites in urine [21, 22]. Most identified correlation were positive suggesting that the body actively metabolizes the parent compound and excretes metabolites in urine. However, only limited data have compared both parental compounds and metabolites in urine. In theory, there should be a negative correlation between the original form and the metabolite form, meaning one variable increases as the other decreases [23, 24]. However, only a positive correlation was found between the urinary TBEP and its metabolite DBEP in our results while no correlation was observed in other OPFRs. There are several points could be applied for explanation: 1) Human OPFRs metabolism must go through multi-step complicated processes including hydrolysis, oxidation, and conjugation, leading to the formation of a diverse range of metabolites. 2) The metabolic pathways of OPFRs can vary depending on the specific compound and the individual’s genetic makeup, age, hormone function, and other unknown factors. 3) Bioaccumulation potentials of OPFRs may be different based on the diverse absorption pathway such as dermal absorption, dust inhalation, or dietary ingestion [25]. 4) The metabolism of OPFRs may also take place in the environment through biodegradation, photodegradation, and hydrolysis, indicating that these metabolites can directly enter human body without via the original form [26]. Due to the above variables, the positive/negative correlation could reflect a detection discrepancy between theoretical and practical measurements in these urine samples.

There were some studies investigating the correlation between lifestyle behaviors and OPFRs exposure. Gibson et al. conducted a prospective study and assigned participants to handwashing and house cleaning intervention for 1 week. Urine OPFRs concentrations were measured at baseline and after the intervention. The study found that increased handwashing with soap and water together with a house cleaning for 1 week was associated with a 50% decrease in OPFR levels in urine samples [27]. Results of this study, via a survey of regular handwashing behavior by questionnaire analysis, demonstrated similar findings. In our results, regular handwashing alone, without additional house cleaning, did not appear to have any impact on OPFR levels. However, we found an association between aging and higher urine BDCPP level, which could be contributed by the followings. First of all, older adults generally had longer exposure periods than younger individuals while older adults may have less efficient metabolism and excretion. Furthermore, older adults may stay indoors longer where the levels of OPFRs are typically higher than outdoors [28]. All of these factors could contribute to an increased accumulation of OPFRs in the bodies of older adults over time. Another interesting finding in our study is that individuals with more meals eaten out and less chilled-ready meals behavior tended to have higher urine BDCPP level. Up to date, the direct relationship between meals eaten out or chilled-ready meals and OPFRs exposure remains largely unexplored. The causal relationship should be very complicated depending on the frequency of meals eaten out, types of food consumed (food packaging and processing equipment) and the settings (types of cooking utensils) in which they are consumed. Further study is needed for clarification and the findings may be helpful for making public health policies and regulations and promoting healthier eating habits.

However, this study has several limitations. First, whether exposure to OPFRs actually causes cancer is still inconclusive and we could not eliminate the possibility that the observed relationship is due to other factors. Secondly, our sample size is not large enough to reach a true association between exposure and outcome. Thirdly, urinary concentrations of OPFRs may not necessarily reflect long-term exposure to these compounds. Additionally, exposure to OPFRs can occur through various sources, including consumer products, food, and the environment, which can make it challenging to accurately assess exposure levels. Lastly, there was no data on mechanisms available.

## Conclusions

This study is the first to demonstrate that EC patients had significant higher urine BDCPP concentration, and this higher level was related to aging, more meals eaten out and less chilled-ready meals consumption behaviors. However, the direct link between lifestyle behaviors and EC remains unclear, with limited clinical evidence. Furthermore, the metabolism of OPFRs in the human body after exposure is highly complex, the lack of a negative correlation between the parent compounds and their corresponding metabolites in urine suggests that some metabolic processes might also occur in the environment. These findings highlight the need for further research to better understand the mechanisms underlying the carcinogenic effects of TDCPP/BDCPP, and to develop strategies to reduce human exposure to these harmful chemicals.

## Supplementary information

**Additional file 1:** OPFRs questionnaire.

**Additional file 2:** The internal standards.

## Declarations

### Availability if data and material

The data that support the findings of this study are available from the corresponding author upon reasonable request.
