# Supplementary material for: Lifestyle factors and urine levels of organophosphorus flame retardants in endometrial cancer: insights from a case-control study
Source: Environ Health Prev Med. 2024 Nov 9;29:63. doi: 10.1265/ehpm.24-00175 (PMC11570647; doi:10.1265/ehpm.24-00175)
Supplement: Supplementary file 1 — Additional file 1: OPFRs questionnaire. [file ehpm-29-063-s001.docx]

| To comply with SPSS computations, multiple-choice questions were categorized into dichotomy variables (0, 1), representing 'categories'. | | |
| --- | --- | --- |
| Age (years) | 0：age ≤ 40 | 1：age > 40 |
| BMI (body mass index, kg/m^2^) | 0：BM ≤ 24 | 1：BMI > 24 |
| Alcohol drinking behavior | 0：none | 1：at least 1 cup daily |
| Meals eaten out per week | 0：≤ 7 times | 1：> 7 times |
| Regular handwashing before meal | 0：none/seldom | 1：often/must |
| Use of plastic food containers | 0：none/seldom | 1：at least once daily |
| Chilled-ready meals consumption | 0：none/seldom | 1：at least once daily |
| Frequent seafood consumption | 0：none/seldom | 1：at least once daily |
| Frequent meat meals consumption | 0：none/seldom | 1：> 0.5 palm size daily |
| Urinate times daily | 0：≤ 5 times | 1：> 5 times |
| Occupational exposure | 0：low-risk | 1：high-risk^a^ |
| Chronic disease^b^ | 0：no | 1：yes |
| Long-term medication^c^ | 0：no | 1：yes |
| Family history of cancer^d^ | 0：no | 1：yes |
| Family history of chronic disease^e^ | 0：no | 1：yes |

^a^ High-risk occupations: defined as working in furniture, electronics, textile manufacturing, recycling facilities, or as a firefighter.

^b^ Chronic diseases were defined as conditions persisting for over 3 months and requiring treatment, including diabetes, hypertension, hyperlipidemia, heart disease, kidney disease, chronic liver disease, and others.

^c^ Participants on medication for over 3 months.

^d^ Cancer family history assessment includes third-degree relatives. The types of cancer considered include brain, oral, nasopharyngeal, esophageal, stomach, liver, pancreatic, bile duct, colorectal, bladder, cervical, ovarian, prostate, bone, breast, lung, laryngeal, thyroid, hematologic malignancies, throat, ureteral, kidney, and intestinal cancers.

^e^ Family history of chronic disease assessment includes third-degree relatives.
