# Supplementary material for: Lifestyle factors and urine levels of organophosphorus flame retardants in endometrial cancer: insights from a case-control study
Source: Environ Health Prev Med. 2024 Nov 9;29:63. doi: 10.1265/ehpm.24-00175 (PMC11570647; doi:10.1265/ehpm.24-00175)
Supplement: Supplementary file 2 — Additional file 2: The internal standards. [file ehpm-29-063-s002.docx]

The internal standards, including TnBP-d27, DNBP-d4, TCEP-d12, BCEP-d8, TBEP-d27, DBEP-d4, TPHP-d15, DPHP-d10, TDCPP-d15 and BDCPP-d10 were purchased from Toronto Research Chemicals Inc. (Toronto, ON, Canada). The concentrations of OPFRs were quantified using the 12-point calibration curves (ranged from 0.02 ppb to 50 ppb with a two-fold dilution) and their recoveries were performed using the internal standard method based on individual isotope-labeled internal standards.

| Compound | Abbreviation | Brand | Product Code | Concentration |
| --- | --- | --- | --- | --- |
| Tri(n-butyl) phosphate | TNBP | Toronto Research | P359445 | 5 g |
| Isotope-labeled | TNBP d27 | Toronto Research | P359447 | 25 mg |
| Tris(2-butoxyethyl) phosphate | TBEP | Toronto Research | T875030 | 10 g |
| Isotope-labeled | TBEP d27 | Toronto Research | T875032 | 2.5 mg |
| Tris(1,3-dichloro-2-propyl)phosphate | TDCPP | SIGMA-ALDRICH | 32951 | 100 mg |
| Isotope-labeled | TDCPP d15 | Toronto Research | T876307 | 2.5mg |
| Diphenyl phosphate | DPHP | Toronto Research | D492000 | 5 g |
| Isotope-labeled | DPHP d10 | Toronto Research | D492002 | 1 mg |
| Triphenyl phosphate | TPHP | Toronto Research | T808990 | 1 g |
| Isotope-labeled | TPHP d15 | Toronto Research | T808992 | 2.5 mg |
| Bis(2-chloroethyl) phosphate | BCEP | Toronto Research | C366790 | 25 mg |
| Isotope-labeled | BCEP d8 | Toronto Research | C366792 | 1 mg |
| Bis(1,3-dichloro-2-propyl) phosphate | BDCPP | Toronto Research | B419095 | 100 mg |
| Isotope-labeled | BDCPP d10 | Toronto Research | B419097 | 1 mg |
| Bis (butoxyethyl) Phosphate | DBEP | Toronto Research | B415125 | 100 mg |
| Isotope-labeled | DBEP d8 | Toronto Research | B415127 | 1 mg |
| Di-n-butylphosphate | DNBP | Dr. Ehrenstorfer GmbH | C 12256000 | 100 mg |
| Isotope-labeled | BNBP d18 | Toronto Research | D429522 | 5 mg |
| tris (2-chloroethyl) phosphate | TCEP | Toronto Research | T875500 | 1 g |
| Isotope-labeled | TCEP d12 | Toronto Research | T875502 | 2.5 mg |
